# Advances and Future Challenges in Microbial Food Safety: Volume I

**DOI:** 10.3390/foods14132231

**Published:** 2025-06-24

**Authors:** Maria Lavilla, Félix Amárita

**Affiliations:** AZTI, Food Research, Basque Research and Technology Alliance (BRTA), 48160 Derio, Spain; famarita@azti.es

## 1. Introduction

Consumers’ growing demand for products with adequate nutritional, physicochemical, and sensory characteristics has challenged both the food industry (including the food production sector) and researchers, necessitating a constant effort in the development of innovative strategies to manufacture new products with optimal characteristics that ensure food safety at the same time [1,2]. However, microorganisms can adapt, survive, and even grow in diverse harsh food environments, and consequently, there is still a considerable burden of foodborne illness, in which microorganisms play a prominent role [3]. Thus, ensuring the microbiological safety of food is a complex issue of continuous concern.

Recent advances in food production and processing technologies, as well as rapid detection methods, are key factors limiting foodborne disease emergence [4,5,6,7]. The development and application of novel preservatives and natural antimicrobials for the biocontrol of foodborne pathogens are also under study [8,9]. Therefore, it is necessary to establish reference publications to show the most recent ground-breaking developments, potential applications, and future trends that complement those already in use, in order to spread the advances in the field and provide alternative tools for the management of microbial food safety throughout the entire food system [10].

To contribute to this knowledge, with new developments in microbial monitoring and novel strategies for pathogen control and inactivation (e.g., technologies, natural preservatives, and hurdle strategies), we present this Special Issue entitled “Advance and Future Challenges to Microbial Food Safety”.

Among the 10 published papers, three main research topics have been covered: (i) innovative treatments and natural strategies for microbial inactivation and control; (ii) the incidence of drug-resistant bacteria in foods; and (iii) novel detection methods for the identification of relevant microorganisms. Also, three reviews are presented.

## 2. Pathogen Inactivation

There are four papers on the first topic, dealing with the effects of diverse non-thermal treatments and natural antimicrobials for pathogen inactivation (contributions 1–4). The first study is an outstanding research article (featured paper) by Pinto et al. (contribution 1) about the inactivation and development control via hyperbaric storage (HS) of *Byssochlamys nivea* ascospores at room temperature (RT: 18–23 °C). *B. nivea* is an extremely heat-resistant form of fungus that may produce mycotoxins and is often associated with the spoilage of heat-processed products. With its previously demonstrated effectiveness in inactivating endospores from other microorganisms, it is of interest to evaluate the feasibility of HS to control the germination and development of ascospores. Based on their results, the authors demonstrated that HS/RT is a safe food preservation methodology, as it enhances ascospore inactivation (up to at least 4.73 log units in 80 °C/30 s pasteurized samples). Also, this technology was able to inhibit ascospore development in samples without a pasteurization step and those pasteurized at 70 °C/30 s, contrarily to samples stored at AP/RT and in refrigeration conditions. As HS/RT effectively prevented the germination and growth of ascospores by preventing the creation of hyphae, this technology is accordingly useful for increased food safety due to its prevention of mycotoxin production.

The second study, by Buitimea-Cantúa et al. (contribution 2), determined the effects of high hydrostatic pressure (HHP) and a pulsed electric field (PEF), two non-thermal technologies for microorganism inactivation, on black and red raspberry juice stabilization. The authors evaluated the inactivation of molds and yeasts and pectin methyl esterase (PME) activity, as well as other important aspects, such as physicochemical parameters (pH, acidity, total soluble solids, and water activity), vitamin C, and phenolic compound content (TPC). Results showed that the observed effect was dependent on the HHP and PEF treatment applied. Also, different effects were observed depending on the considered microorganism and phytochemical compound: Overall, both technologies achieved significant inactivation rates of molds (up to 3.72 log) and yeast (up to 3.19 log). Additionally, although PME activity was insufficiently inactivated (22% inactivation at the most), an increment in the TPC and vitamin C was observed in both the HHP- and PEF-treated juice. As both phytochemical compounds are associated with a fresh flavor, the authors concluded that HHP and PEF are valuable technologies for increasing the microbiological and nutritional quality of black/red raspberry juice.

The third study in this topic, by Sawangrat et al. (contribution 3), dealt with the application of plasma-activated water (PAW) generated from pinhole plasma jets for pesticide degradation and microorganism decontamination in chili (*Capsicum annuum* L.) in comparison to other treatments (i.e., deionized water (DI), ethanol, and NaClO). The mechanisms of PAW treatments on pesticide residues, spoilage, and pathogenic microorganisms in both solution and chili were also investigated. It was confirmed that PAW was effective in decontaminating both pesticides and microorganisms of health concern. Also, PAW showed higher efficiency compared to other treatments in controlling Anthracnose (a chili disease caused by the fungus *Colletotrichum gloeosporioides*) via 100% inhibition of fungal spore germination. Although further improvements to the plasma system are needed to support its application for industrial and consumer use, this technology could be easily adopted in the production line as a sanitizer to prepare raw materials (i.e., fruits and vegetables).

The fourth original study in this topic, by Kitsiou et al. (contribution 4), explores the antimicrobial efficacy of grape seed extract (GSE) as a natural antimicrobial against foodborne bacterial pathogens. Systematic quantitative monitoring of the microbial dynamics/kinetics over time was used to better determine its microbial inhibitory effects against three important foodborne pathogens and their stress mutants in different growth phases and at different inoculum levels. In general, the Gram-negative bacteria under study (*Escherichia coli* and *Salmonella* Typhimurium) were less susceptible to GSE compared to *Listeria monocytogenes*. GSE was found to be highly effective in inactivating *L. monocytogenes*, with higher inactivation achieved for higher GSE concentrations and lower initial inoculum levels. Also, generally, stationary phase cells were more resistant/tolerant to GSE compared to exponential phase cells (for the same inoculum level). These studies concurrently emphasized the potential of novel sustainable technologies and antimicrobial strategies in the food industry against common food-related pathogens.

## 3. Multidrug-Resistant Microorganisms in Foods

There are two manuscripts in this Special Issue focused on the second topic: The incidence of multidrug-resistant microorganisms in foods (contributions 5, 6). Antimicrobial resistance is a growing global problem that poses a serious threat to human health. Food may be an important route of exposure to antibiotic-resistant bacteria, which significantly increases concerns regarding microbial safety in the food chain. In this sense, in the first study on this topic, Abo-Almagd et al. (contribution 5) determined the prevalence, antibiotic resistance profiles, phylogroups, and β-lactamase genes of *E. coli* isolates from chicken carcasses marketed in Egypt. The study reported the high contamination rates (98%) of chicken carcasses with *E. coli*. Furthermore, 85 strains of 425 isolates were studied for antimicrobial resistance profiles, phylogroups, and β-lactamase genes. The results showed, in general, a significant rate of multidrug resistance (89.4% of strains tested against 24 different antibiotics). More specifically, 22 (25.88%) isolates harbored bla_CTX-M_ and were resistant to ampicillin, cefazoline, and ceftriaxone, and nine (10.59%) *E. coli* strains harbored AmpC- β-lactamase bla_CMY_ and were resistant to ampicillin. Although most of the isolated strains were commensal *E. coli*, these results highlight the antimicrobial resistance problem in Egypt, and the likely potential dissemination of transmissible resistance mechanisms among foodborne pathogens in the food chain.

The second paper, by Akaeze et al. (contribution 6), looked into the contamination of watermelons with drug-resistant pathogenic *Enterobacteriaceae* strains, and the potential differences between organic and conventional cultivation. *Enterobacteriaceae* strains were isolated and tested for antimicrobial resistance against 12 common antibiotics. Findings from this study confirm the presence of antibiotic-resistant *Enterobacteriaceae* strains in organic watermelons in Nashville, although further research should be directed towards investigating the genotypic aspects of antibiotic resistance in the isolates. Both works on this topic show the need to monitor and control the use of antibiotics, as well as to establish continuous epidemiological studies for the rapid detection of threatening resistances in the food chain.

## 4. Pathogen Detection and Identification

One original paper has been published that addresses the third topic, dealing with the development of novel detection methods for the identification of relevant microorganisms and its impact on food safety. Since some fungi are potential mycotoxin producers, it is necessary to detect and identify the fungal community during storage to ensure its adequate quality and safety. In this paper by Wang et al. (contribution 7), a rapid multiplex PCR is described for distinguishing closely related *Aspergillus* species in tea. The primers designed, based on orphan gene sequences, allowed researchers to identify those species with a proven absence of common mycotoxin biosynthetic gene clusters: *A. cristatus*, *A. chevalieri*, and *A. pseudoglaucus*. Also, this paper opens up new opportunities for the future development of new species-level identification methods based on the strategy of orphan gene sequence specificity.

## 5. Reviews

Three reviews have been published in this Special Issue, collecting and summarizing the most recent research on three important topics related to microbial food safety. The first work, by Chen et al. (contribution 8), reviews the current progress in research on actinomycetes (a group of filamentous bacteria) related to Baijiu fermentation. This review includes research about actinomycetes’ isolation and identification, distribution, interspecies interactions, systems biology, and their main metabolites and microbiome applications during Baijiu fermentation, providing the unique flavor of Baijiu, which is distinct from that of other distilled liquors. Also, actinomycetes produce a large amount and diversity of antibiotics, such as heptaene macrolide antibiotics and streptomycin, which can inhibit the growth of fungi and pathogenic bacteria, also increasing beverage safety.

On the other hand, Fischer et al. (contribution 9) published the second review, looking over the use of protective cultures as a simpler and more cost-effective alternative for preserving food products, guaranteeing food safety, and prolonging shelf life. Contrarily to chemical preservation (which changes texture and taste) and physical preservation (which requires equipment and energy), biological preservation can be easily achieved through food fermentation via microorganisms such as yeasts and lactic acid bacteria (LAB), which leads to new, often very tasty food products. In this review, the authors report the application of LAB in bio-protection, not only in fermented products, but also in varied perishable foods such as smoked salmon, minced meat, salad, and sprouts, noting a significant increase in shelf life and food safety without affecting food taste or texture. The authors also describe the characteristic features of protective microorganisms regarding the underlying mode of action of antibacterial mechanisms. Ultimately, this review provides an important and comprehensive guideline for the use of protective cultures by offering strategies for strain identification and validation, providing important information about market and legal restrictions.

The last review, by Lavilla et al. (contribution 10), deals with the use of bacteriophages (phages), viruses of bacteria, as new effective biocontrol and bio-protection agents for increased food and water safety, or as natural preservatives to extend product shelf-life. Phages are potent antimicrobials against most pathogenic bacteria and can be usefully implemented for numerous environmentally friendly applications at each stage of the farm-to-fork continuum, within the One Health concept. Accordingly, this review summarizes the latest research on the use of these microorganisms to control *Campylobacter*, *Listeria monocytogenes*, *Salmonella*, and *Vibrio* spp., from animal production (especially broilers and aquaculture) to the post-slaughtering stage of different food products (poultry meat, beef, eggs, fish, and dairy…). Importantly, in order to assist future phage-based real-world applications, legal compliance and pending research issues to be diffused by forthcoming studies are also taken into account.

## 6. Conclusions

In summary, the 10 papers published in this Special Issue are a strong representation of the research activities and works addressing advances in tackling current and future challenges in microbial food safety. Most authors who contributed to this issue concluded that further research in their topics is required to optimize the conditions of non-thermal treatments and detection methods, and encourage novel natural bioactive compounds for feasible foodborne pathogen inactivation.
